# Toward In Vivo Cancer Detection: X-Ray Scattering on Thick Phantom Samples

**DOI:** 10.3390/molecules30081655

**Published:** 2025-04-08

**Authors:** Viacheslav Kubytskyi, Masroor Khonkhodzhaev, Aika Tanaka, Audrey Nguyen, Alexander Lazarev, Byron Aram, Keith Rogers, Lev Mourokh, Pavel Lazarev

**Affiliations:** 1 Matur UK Ltd., 5 New Street Square, London EC4A 3TW, UKplazarev@eosdx.com (P.L.); 2 Laboratoire de Physique des 2 Infinis Irène Joliot-Curie, UMR9012, CNRS, Université Paris-Saclay, Bât. 209, 91405 Orsay, France; 3 Physics Department, Queens College, City University of New York, 65-30 Kissena Blvd, Flushing, NY 11367, USA; 4 EosDx, Inc., 1455 Adams Drive, Menlo Park, CA 94025, USA; atanaka@eosdx.com (A.T.); anguyen@eosdx.com (A.N.); baram@eosdx.com (B.A.); k.d.rogers@cranfield.ac.uk (K.R.); 5 Shrivenham Campus, Cranfield University, Swindon SN6 8LA, Wiltshire, UK

**Keywords:** cancer detection, X-ray scattering, Monte Carlo simulations, structural biomarkers, lipid molecules

## Abstract

As the number of new breast cancer cases grows around the world, there is an unmet need for fast, accurate, and low-cost methods of early cancer detection. It was previously shown that X-ray scattering on lipid molecules can provide the necessary structural biomarker. However, these measurements were performed on small ex vivo samples, and to ensure the progress to in vivo diagnostics, the approach should be extended to larger tissues. We use the phantom fat samples to establish such a procedure. In the obtained X-ray scattering patterns, we observe the characteristic features for the inter-fatty-acid molecular distance. The large size of the samples led to the peak broadening; however, the features remain visible up to 10 cm in thickness. The experimental data are in excellent agreement with the Monte Carlo simulations based on the form factors obtained from the small samples. Our results usher the way for the in vivo monitoring of the structural biomarkers of breast cancer.

## 1. Introduction

Breast cancer is the most common cancer diagnosed among females in the United States. Approximately 1 in 8 women in the United States (13.1%) will be diagnosed with invasive breast cancer, and 1 in 43 (2.3%) will die from the disease [1]. In 2024, an estimated 310,720 new cases of invasive breast cancers and 56,500 new cases of ductal carcinoma in situ will be diagnosed among women and 2790 will be diagnosed among men in the United States [1]. The mortality rate has slightly decreased over the last few years [2], which can be attributed to progress in early diagnostics [3,4]. However, the total number of cases and mortality are still worryingly high; more breakthroughs are needed.

Mammography is the current standard breast screening technique but has apparent limitations [5]. It is less effective for women with dense breasts, fails to detect 10–30% of breast cancers [6], exposes patients to radiation, and often leads to false positives and overdiagnoses, which cost USD 4 billion annually [7]. This also requires additional screening tests (with a total cost of USD 7.91 billion [8]); the sequence of procedures usually takes several weeks with multiple appointments. Moreover, biopsies are often essential for conclusive diagnoses. These involve histopathological analysis and genetic expression profiling, making the process expensive, time-consuming, and discomforting for patients [9]. It should be noted that over 80% of patients undergoing biopsies do not have cancer, rendering many of them unnecessary [10]. Ultrasound, often used for dense breasts as complementary to the mammogram, while non-invasive, has lower sensitivity and higher variability in diagnosis [11]. Although advanced techniques, such as 3D mammograms (tomosynthesis), molecular breast imaging, computerized tomography, magnetic resonance imaging, contrast-enhanced digital mammography, and positron emission tomography, offer more accurate diagnostics, they are more costly and unsuitable for mass screening [5].

Instead of direct probes or imaging of suspicious abnormalities, cancer detection research has recently focused on biomarkers [12,13,14,15], i.e., precancerous or cancer-induced biochemical or molecular alterations. Specific breast cancer-associated biomarkers comprise macromolecules such as nucleic acid (DNA/RNA), proteins, and intact cells [16]. Another prominent biomarker approach is the liquid biopsy, which detects circulating tumor DNA and cells in body fluids [17,18]. The biomarker tests are non-invasive and performed ex vivo, addressing the convenience and comfort of patients. Still, they are relatively expensive and a significant amount of time is required to process the results.

Another approach for breast cancer detection employs *structural* biomarkers obtained from X-ray scattering experiments with human breast tissues. Small-angle X-ray scattering (SAXS) addresses the breast cancer-induced modifications of collagen fibril repeat distances [19,20,21,22] and changes to the associated amorphous scattering [21,22,23,24]. Wide-angle X-ray scattering (WAXS) delivers information about variations in the lipid and aqueous components [25,26,27,28,29,30,31,32]. In particular, it was shown [28,32] that in cancerous tissues, the intensity of a peak at approximately *q* = 14 nm^−1^ is reduced (where *q* is momentum transfer measured in reciprocal space units). This intensity maximum arises from inter-fatty-acid molecular distances. Cancer cells modify lipid metabolism [33,34,35], activating, desaturating, or elongating fatty acids. Moreover, they synthesize de novo lipids that can differ from those in circulation. The suppression of the 14 nm^−1^ peak indicates such changes. Concurrently, an intensity maximum at approximately *q* = 20 nm^−1^ (associated with the oxygen–oxygen distance in the tetrahedral structure of water) increases. This effect can serve as a structural biomarker for breast cancer detection; however, the experiments mentioned above are primarily performed at synchrotron facilities with a limited number of samples. Recently, we employed bespoke *laboratory* diffractometers for the same purposes and obtained the same peak variations [36,37] on a large number of samples both in the United States [36] and the United Kingdom [37]. For the WAXS measurements, we achieved 96.3% sensitivity and 91.6% specificity for binary cancer/non-cancer differentiation of the samples using the machine learning approaches.

The proposed cancer detection approach based on X-ray diffractometry appears promising as it is non-invasive, rapid, and relatively inexpensive. However, significant challenges must be overcome before it can be used for in vivo applications. In particular, previous experiments were performed on small samples a few millimeters thick; it is unclear if the same observations can be made for samples that are several orders of magnitude larger. First, the incoming X-ray photons can be mostly absorbed and the scattered signal would be weak. Second, the photons can be scattered multiple times within an extended tissue, resulting in scattering patterns that may be difficult to interpret. Third, photons scattered by the same structures at different positions along the thick sample will intersect the detector at different positions, leading to peak broadening. Confounding this further, every detector pixel may also receive photons scattered through a different angle when the scattering source occurs at different positions along the primary beam.

The present paper addresses these issues by performing X-ray scattering experiments on phantoms constructed from pork-neck bone ends. The absorption and scattering cross-sections of these and breast tissue are similar. Given that there is a lower proportion of fat content in the pork neck, it is an appropriate tissue with which to challenge our approach. Instead of the copper anode of Refs. [36,37], we used a silver anode with a shorter wavelength, leading to a significantly larger penetration depth. Although this initial work focuses on the ability of our system to detect specific scattering maxima, the full measured range of scatter could also potentially be employed. Two device configurations were used, with and without a beam stop, which prevents the detector saturation and pixel crosstalk caused by the unscattered X-ray beam. We supported our experiments with Monte Carlo simulations described in the Methods section below.

## 2. Results

### 2.1. Measurements of X-Ray Scattering

**Figure 1:**
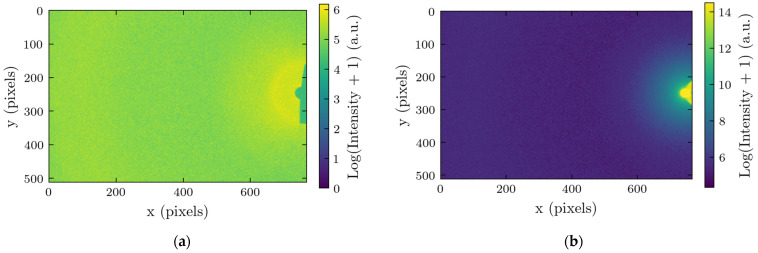
Obtained X-ray scattering images with the intensities in the logarithmic scale. Numbers indicate the position of the pixels on the detector: (**a**) device with the beam stop; and (**b**) device without the beam stop.

X-ray scattering experiments were performed on phantom fat samples of various thicknesses—2, 4, 6, 8, and 10 cm—to evaluate the role of sample thickness on the characteristics of the scattered signal. The measurements were taken at three lateral points on each sample. The representative images obtained at a thickness of 2 cm are shown in Figure 1 for two device configurations, with and without the beam stop.

**Figure 2:**
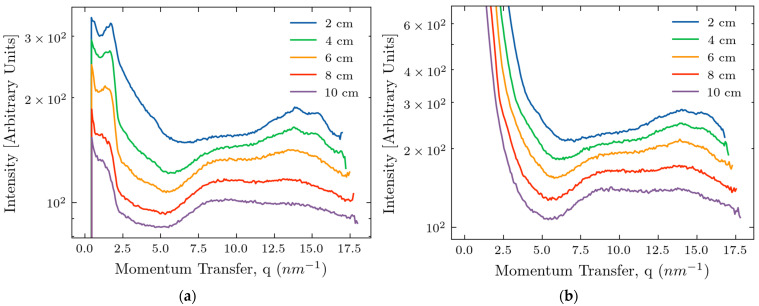
Dependencies of the scattered intensities on the transfer momentum for various sample thicknesses: (**a**) device with the beam stop; and (**b**) device without the beam stop.

After the azimuthal integration and averaging over three points of the measurements, we obtain the dependence of intensity on the momentum transfer, as shown in Figure 2, for the two device configurations.

An intensity maximum, characteristic of fat, occurring at approximately 14.5 nm^−1^, is clearly seen in all curves, even for the samples with a thickness of 10 cm. During this work, we focused on this intensity maximum as it is a point of reference reliably associated with the tissue composition. At smaller thicknesses, this peak is resolved into several narrower maxima associated with different forms of lipids [38,39]. At larger thicknesses, inherent peak broadening causes the peaks to overlap. The intensity of the scattered signal decreases with the increasing thickness, *d*, because of the dominance of X-ray absorption (*d* > 1/*μ* where *μ* is the tissue linear absorption length at Ag wavelengths). At high *q* values, the signal-to-noise ratio is lower without the beam stop, making the beam stop desirable for detecting the fat peak at 14.5 nm^−1^. However, at small values of *q*, the beam stop produces artifacts, as seen in Figure 2a, making the analysis of the signals difficult. In the region below 5 nm^−1^, signal decay with 1/*q* dependence is associated with X-ray scattering by air molecules in the interval between the sample and the detector. Any amorphous scattering with a 1/*q*^4^ dependence overlaps with the primary beam or is screened by the beam stop and is not apparent. A low-intensity peak at 4.5 nm^−1^, representing the third order of the triglyceride packing [38,39], is also visible. There is a peak or shoulder at about 9 nm^−1^; however, the origin of this feature is unclear. We can speculate that it comes from the connective tissues.

**Figure 3:**
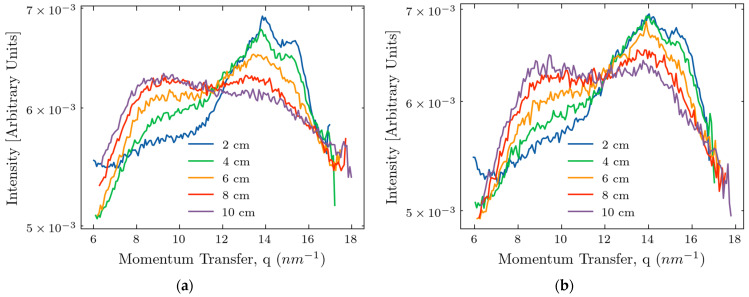
Normalized dependencies of the scattered intensities on the transfer momentum for various sample thicknesses: (**a**) device with the beam stop; and (**b**) device without the beam stop.

To make the peaks at larger thicknesses more pronounced, we normalized the curves for the *q*-range between 6 and 18 nm^−1^ and present them in Figure 3.

**Figure 4:**
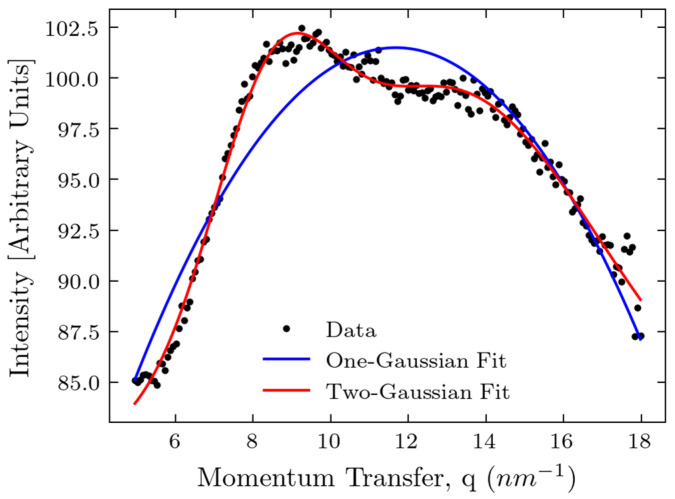
Gaussian fit for the 10 cm thickness curve: one-Gaussian vs. two-Gaussian fitting.

With the thickness increasing, the magnitude of the peak at 9 nm^−1^ increases; it starts to overlap with the peak at 14 nm^−1^. Probably, it is related to the increased content of connecting tissues in the large phantom samples. To prove the visibility of the 14 nm^−1^ peak at thicknesses of 10 nm, we performed the automated Gaussian peak fitting shown in Figure 4. For both models, a constant background term was included. The curve fitting was performed using the ‘SciPy’ Python package, version 1.15.0; the algorithm converged successfully.

It is evident from this figure that the two-Gaussian fit is much better; the two peaks can still be separated. To support the visual impression, we calculated the coefficient of determination (R2) and root mean squared error (RMSE). The one-Gaussian fit resulted in R2 of 0.874 and RMSE of 1.774 arbitrary units, while the two-Gaussian fit resulted in R2 of 0.985 and RMSE of 0.610 arbitrary units, demonstrating the superiority of the two-Gaussian fit and the existence of the peak at 14 nm^−1^.

### 2.2. Monte Carlo Simulations

Monte Carlo simulations were performed to complement the experimental results and provide a theoretical understanding of the scattering behavior in thick samples. The simulations allowed for the independent control of parameters, such as scattering type, photon energy, and sample geometry, offering insights that are otherwise extremely difficult to obtain experimentally. In this model, the monochromatic photons were emitted from the source with a specific geometry. The tissue was represented by material with form factors corresponding to adipose tissues [40]. Each photon from the source has a certain probability of being absorbed, transmitted, or scattered (via the photoelectric, Rayleigh or Compton process) one or more times within the sample. Depending on the geometry, some scattered photons will not intersect the detector. For each X-ray photon the detector captures, the following information was stored for analysis: energy; six-dimensional coordinates for position and momentum; ID for the event; the number of Rayleigh scatterings; the number of Compton scatterings; and the number of diffraction events. We consistently used 5 × 10^7^ incident photons for our simulations.

**Figure 5:**
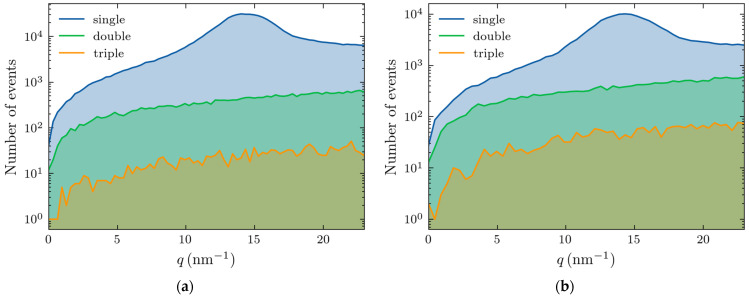
Number of X-ray photons (out of 50 million) experiencing single, double, and triple scattering within the adipose sample with: (**a**) 4 cm thickness; and (**b**) 10 cm thickness.

Higher-order scatterings—double, triple, and beyond—become increasingly prominent with increases in thickness. The photon numbers for these events (out of 5 × 10^7^) are shown in Figure 5, with the left and right panels corresponding to thicknesses of 4 and 10 cm, respectively.

For a thickness of 4 cm (Figure 5a), the scattering accounts for 83% of the total signal, whereas in a 10 cm sample, this is reduced to 64%. The triple scattering is rare in both cases, appearing as a background noise, and the adipose peak is well resolved.

**Figure 6:**
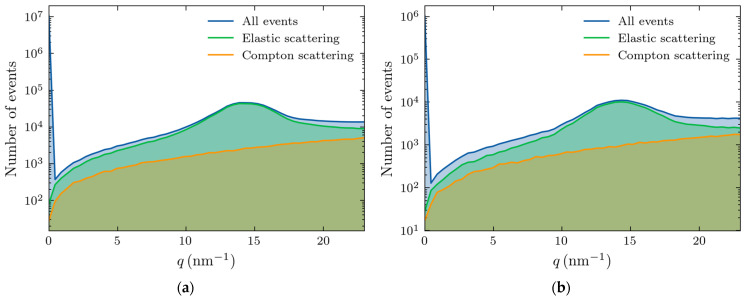
Number of X-ray photons (out of 50 million) experiencing elastic (Raleigh) or Compton scattering within the adipose sample with: (**a**) 4 cm thickness; and (**b**) 10 cm thickness.

We also separated the contributions of various scattering processes, with the results shown in Figure 6 for thicknesses of 4 (left panel) and 10 cm (right panel).

This figure demonstrates that the Raleigh scattering dominates the region of interest in our studies (10–20 nm^−1^), especially at the momentum transfer of the adipose peak. The large feature at zero transfer momentum corresponds to the unscattered photons. The magnitude of the signal is much smaller at a thickness of 10 cm because of increased absorption.

### 2.3. Comparison of Experiments and Simulations

**Figure 7:**
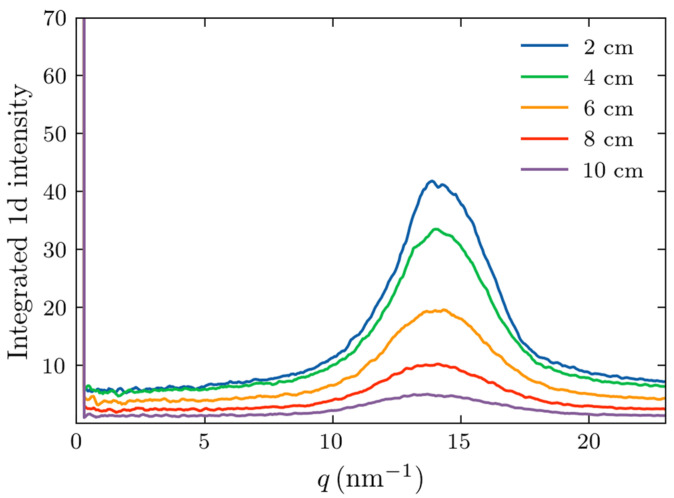
Simulated dependencies of the X-ray scattering intensity on the transfer momentum for various thicknesses of the fat samples.

To compare the Monte Carlo results with the experimental data, we simulated X-ray scattering on adipose samples with thicknesses corresponding to those of the experimental counterparts. In all cases, 5 × 10^7^ photons were incident on the sample. The scattered photons incident on the detector were azimuthally integrated to provide the dependence of the intensity on momentum transfer, *q*. The results are shown in Figure 7.

**Figure 8:**
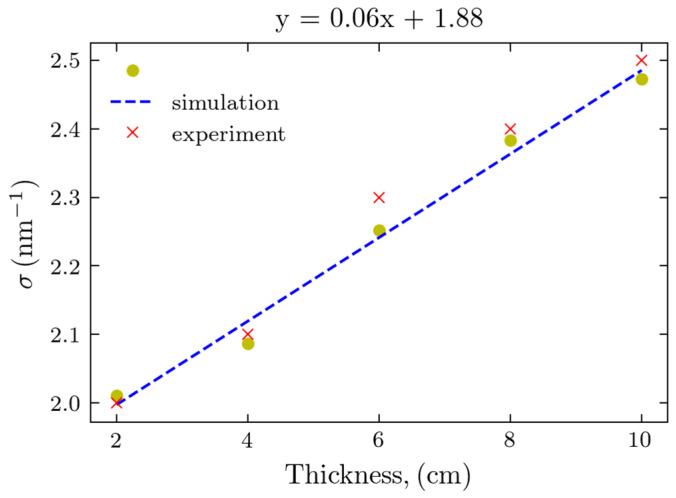
Calculated peak widths of the Gaussian peaks for experimental and simulated curves and the linear fit for the simulations.

It is evident from Figure 7 that the fat form factor produced a peak at approximately 14.5 nm^−1^, as expected. The width of this peak increased with increasing thicknesses and its magnitude decreased. However, the peak remains discernable up to a thickness of 10 cm, similar to that of the experimental observations in Figure 2. To further compare the experimental results and simulations, we performed the least squares, Gaussian fit to the maxima profiles of Figure 7 and Figure 2a. Experimentally, the device with the beam stop was used due to its greater S/N. The determined peak widths of the calculated and experimental Gaussian peaks are presented in Figure 8. This provides an assessment of our geometric modeling (anticipating the impact of sample thickness on peak width) and a validation of the Monte Carlo simulations.

Both results follow the linear fit with the thickness increasing; remarkably, the values obtained from the Monte Carlo simulations are in excellent agreement with those of the actual experiments, as the corresponding points are almost overlapping. This linear relationship is expected from simple geometric considerations. Photons scattered from sample volumes closest to the source will intersect the detector further from the primary beam than those scattered through the same angle but from volumes furthest from the source. The close agreement is also evidence for the validity of the MC modeling.

**Figure 9:**
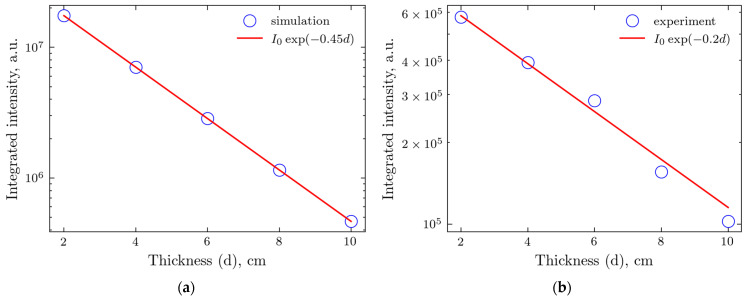
Integrated transmitted intensity for various thicknesses of the samples and the exponential fit: (**a**) Monte Carlo simulations; and (**b**) experiment.

To explore the validity of the intensity changes occurring through the tissue as predicted by the MC model, we also compared the tissue attenuation for simulated and actual experiments by integrating the intensity at small *q*, up to *q* = 3, using Figure 7 for the simulated results and Figure 2b (no beam stop) for the experiment. The results are shown in Figure 9.

Beer–Lambert’s law [41], with its exponential dependence on the thickness, works well for both simulated and experimental data. Still, the attenuation coefficient for the latter is somehow more than two times smaller. This is probably caused by the non-uniformity of phantom packing with the probable inclusion of other tissue components. At the same time, the material for the simulations is supposed to be uniform.

## 3. Discussion

It is evident from Figure 2 that the proposed cancer detection method based on structural biomarkers obtained utilizing X-ray scattering is feasible for in vivo applications. The intensity maxima corresponding to scattering from fat, previously used for cancer/non-cancer classification in small biopsy breast cancer samples, is visible up to a sample thickness of 10 cm. This was achieved due to an updated laboratory diffractometer with an X-ray source based on more penetrating Ag wavelength photons. We explored two device configurations, with and without a beam stop. At large momentum transfer values, the signal produced by the device with the beam stop is less noisy, and evaluating the fat peak properties is easier. However, at small *q*, the beam stop produces artifacts, and the device can better examine the small-angle scattering without it. Application of the approach to clinical applications with sample thicknesses larger than 10 cm might be challenging. Conventional mammography is undertaken with breast compression that reduces the breast thickness to less than 10 cm; thus, our approach would be viable in these circumstances. Alternatively, the clinicians will perform the measurements on the parts of the organ with smaller thicknesses.

Monte Carlo simulations appeared to be a valuable tool for supporting the X-ray scattering experiment. Using the form factor obtained from synchrotron experiments with small samples, we could model the experiment with much larger samples and with different energies of the X-ray photons. The fat peak widths obtained in the simulation are in excellent agreement with the experimental data.

The results obtained in our work demonstrate the potential applicability of in vivo breast cancer detection pending further clinical validation. This approach can be first extended to phantom samples prepared from human breast tissues, and eventually, our diffractometer can be modified to be used in vivo. Monte Carlo simulations can connect the small- and large-sample results with the form factors initially taken as the mixture of the tissue components and ultimately determined from direct X-ray scattering experiments with human tissues.

The proposed X-ray diffraction-based approach is fast, non-invasive, and inexpensive. It will readily complement mammography, providing independent evidence of structural biomarkers of cancer or its absence and eliminating unnecessary biopsies.

## 4. Materials and Methods

### 4.1. Sample Preparation

Three mylar tubes were constructed using stencil sheets, each with a diameter of 4 cm and an initial length of 10 cm. The first tube was filled with adipose tissue isolated from the neck bone ends of pork. The second tube was filled with a silver behenate (AgBH) powder (Thermoscientific^®^ 045494.06, Waltham, MA, USA) as a calibration standard. The third tube was left empty to measure background levels. For each measurement, the sample height was progressively reduced by removing material and trimming the tube in 2 cm increments, starting from the initial length of 10 cm. Measurements were taken at tube lengths of 10 cm, 8 cm, 6 cm, 4 cm, and 2 cm.

### 4.2. XRD Measurements

Measurements were performed using a custom-built, vertical optical axis diffractometer equipped with a Photon III 7HE detector and a silver anode X-ray source built by Bruker Corporation (Billerica, MA, USA). The source produced monochromatic photons using multilayer X-ray mirrors. The Ag source was chosen as a compromise between sufficient photon penetration (22 keV photons have a linear absorption coefficient of 0.16 cm^−1^ in breast tissue) and the geometric arrangement necessary to record the scattering distributions over the range of *q*-space required. The CMT detector featured a 768 × 512 pixel array, with 135-micron pixel size. Experiments were undertaken with and without the use of a primary beam stop. The detector was aligned so that the primary beam would intersect near the center of the shorter side and the edge of the sensitive area, such that a broader range of scattering angles could be measured. The X-ray source power was 50 kV and 1000 mA (50 W), while a standard 110 V outlet powered the entire instrument. The primary beam was shaped and focused by Montel mirror-focusing optics produced by Incoatec (Geesthacht, Germany). Then, the beam was collimated with a custom-built tungsten collimator with an 800-micron diameter pinhole aperture. The use of such a small-area beam and monochromatic photons has the additional benefit of minimizing any radiation damage to the tissues.

Following the background and calibration scans, measurements of animal adipose tissue were conducted. Starting at a sample length of 10 cm, measurements were collected at three distinct points (P1, P2, and P3) on the tissue. For P1, exposure times of 30 s, 60 s, and 120 s were used, while for P2 and P3, a single exposure time of 120 s was applied. After completing measurements at each length, the sample length was reduced by 2 cm and the process was repeated until reaching the final length of 2 cm. The same procedures were applied for scans with a beam stop, including trimming the sample and measuring three distinct points at each length. These measurements were performed using an exposure time of 180 s.

### 4.3. Image Processing

As the beam stop created a shadow region with a low intensity of the same, it was cut out and replaced with “null” values so that the azimuthal integrator would disregard those pixels. In this, the shadow region was manually overlayed. The calibration measurement of AgBH with a thickness of 2 cm was used to determine the location of the beam center and the sample-to-detector distance. The calculated distance to the sample center was 71 cm; therefore, the actual distance to the sample support platform was 72 cm. For samples of different thicknesses, the effective sample-to-detector distances for azimuthal integration were calculated as 72—(thickness/2) cm.

### 4.4. Data Processing

After the azimuthal integration of scattering patterns, the dependence of the intensity on the distance to the center was obtained. It was recalculated to the dependence on the momentum transfer *q* = (4π sin *θ*)/*λ*, where tan 2*θ* is the ratio of the distances from the pixel to the center and from the sample to the detector. Next, the measurements of the three points corresponding to the same distance were averaged.

### 4.5. Monte Carlo Simulations

For Monte Carlo simulations, we used the Geant4 simulation toolkit [42], which is a widely used platform for modeling interactions between particles and matter. Initially developed for high-energy physics applications at CERN, it is now also applied in other fields, including medical physics and materials science, due to its ability to simulate complex geometries and interactions across various energies. Geant4 provides tools with which to define geometries, materials, and physics processes, making it suitable for X-ray scattering simulations. It includes elastic (Rayleigh) and inelastic (Compton) scattering, as well as photoelectric absorption, with the material form factors derived from experimental scattering patterns, mainly measured at a synchrotron. While effective for non-biological materials, this approach is challenging to apply to biological tissues due to their complex and variable compositions. Paterno et al. [40] demonstrated the applicability of this software by simulating SAXS experiments on structured materials. In this, biological samples were approximated as mixtures of four primary components: fat, water, collagen, and calcium hydroxyapatite. The overall form factor of the sample is then calculated as a weighted combination of these basis materials, each incorporating interference effects. In the present work, we used only the fat form factor. We consider a pencil 22 keV X-ray beam (Ag wavelength) with a radius of 100 μm, with the air scattering for the photons leaving the sample being neglected.

## 5. Conclusions

The main conclusion of our work is that the structural biomarkers obtained in the small biopsy samples can be observed in the samples of the sizes of the human organs. This suggests the potential applicability of fast, accurate, and inexpensive cancer detection based on X-ray scattering. Although we are not presenting any in vivo results, we believe that this work presents an essential initial step on the translation pathway toward in vivo cancer detection.

## Data Availability

The files with the XRD patterns and results of Monte Carlo simulations are available at https://doi.org/10.5281/zenodo.14775480 (accessed on 31 January 2025). The codes for the image processing are available upon request.
